# 1991. Cluster of Non-toxigenic *Corynebacterium diphtheriae* Infective Endocarditis Prompting Epidemiologic Investigation — Seattle, Washington 2020-2023

**DOI:** 10.1093/ofid/ofad500.118

**Published:** 2023-11-27

**Authors:** Ellora Karmarkar, Tom Fitpatrick, Talia Himmelfarb, Eric J Chow, Hayden Z Smith, Kristine F Lan, Jason I Matsumoto, H Nina Kim, Paul S Pottinger

**Affiliations:** University of Washington, Seattle, WA; University of Washington, Seattle, WA; University of Washington, Seattle, WA; Public Health - Seattle & King County, Seattle, Washington; University of Washington, Seattle, WA; University of Washington, Seattle, WA; Harborview Medical Center-University of Washington, Seattle, Washington; University of Washington, Seattle, WA; University of Washington School of Medicine, Seattle, WA

## Abstract

**Background:**

Non-toxigenic *Corynebacterium diphtheriae* (*C. diphtheriae*), an aerobic gram-positive bacillus, is often associated with wound infections. However, it can also cause outbreaks and invasive disease including infective endocarditis (IE). After identifying a cluster of *C. diphtheriae* IE cases, we conducted an epidemiologic investigation to identify additional *C. diphtheriae* detections within our university-affiliated hospital system from 2020-2023.

**Methods:**

We performed retrospective chart review of any patients with *C. diphtheriae* detected in a clinical specimen (i.e. wound, blood, sputum) between 9/1/2020 and 4/1/2023. A confirmed case of *C. diphtheriae* IE was defined as at least two positive monomicrobial blood cultures with vegetation(s) on echocardiogram; probable IE was defined as positive monomicrobial blood cultures with embolic phenomena. We describe demographic and clinical characteristics of all patients with *C. diphtheriae* detection, including patients with *C. diptheriae* IE.

**Results:**

Between 9/1/2020 to 4/1/2023, 45 patients (median age 44 years, range 23-75 years; 76% male, 64% non-Hispanic White, 80% unstably housed) had ≥ 1 clinical specimen with *C. diphtheriae;* 5 (11%) patients had confirmed (n=4) or probable (n=1) IE. The largest number of *C. diphtheriae* detections were in December 2022 (Figure 1). Forty-three patients (96%) reported substance use; 19 (42%) reported history of injection drug use (IDU). *C. diphtheriae* was detected in 34 (76%) polymicrobial wound cultures, 7 (16%) polymicrobial blood cultures with concomitant Staphylococcal or Streptococcal bacteremia, 5 (11%) blood cultures from *C. diphtheriae* IE, and one (2%) sputum specimen. The five IE cases (80% male, 60% unstably housed) were diagnosed between 5/2022-3/2023 and had current IDU (60%) or open wounds (40%). Four had embolic phenomena; two received cardiac surgery. Four (80%) patients with IE died: three from IE and one from COVID-19.

**Figure 1:**
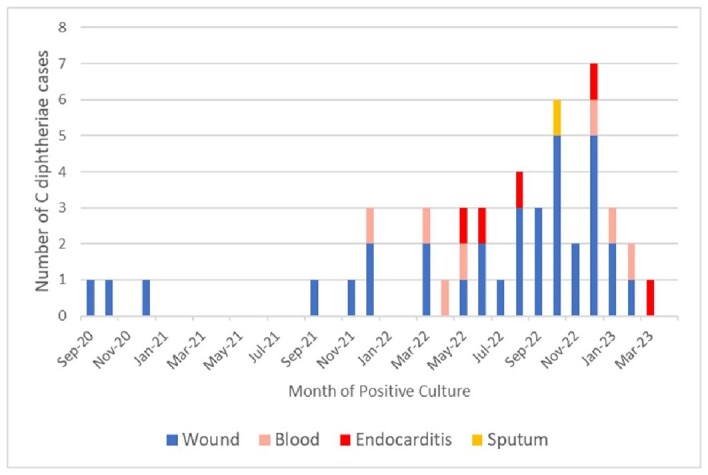
Epidemiologic curve of C. diphtheriae cases in patients with positive wound cultures, blood cultures, endocarditis, and sputum cultures within a university-affiliated hospital system, September 1, 2020-April 1, 2023.

**Conclusion:**

A cluster of *C. diphtheriae* IE cases and rising *C. diphtheriae* detections in our hospital system raise concern for a local outbreak disproportionately affecting patients who use substances and are unstably housed. Outbreak investigation and prevention efforts (wound care, linkage to care) are critical to prevent additional mortality.

**Disclosures:**

**H. Nina Kim, MD, MSc**, Gilead: Grant/Research Support

